# Unusual Presentation of Primary Cardiac Lymphoma in a 61‐Year‐Old Woman: A Case Report

**DOI:** 10.1002/ccr3.70856

**Published:** 2025-09-03

**Authors:** Danial Fazilat‐Panah, Majid Nabipour, Seyed Reza Najafi, Mina Heidarian

**Affiliations:** ^1^ Department of Radiation Oncology, School of Medicine, Shahid Rajayee Hospital Babol University of Medical Sciences Babol Iran; ^2^ Department of Internal Medicine, School of Medicine, Rouhani Hospital Babol University of Medical Sciences Babol Iran; ^3^ Student Research and Technology Committee Babol University of Medical Sciences Babol Iran

**Keywords:** DLBCL, echocardiography, primary cardiac lymphoma, R‐CHOP regimen chemotherapy

## Abstract

Primary cardiac lymphoma (PCL) is one of the rarest primary malignancies of the heart. This report describes a rare presentation of diffuse large B‐cell lymphoma (DLBCL) in a 61‐year‐old woman from Iran who presented with weakness, dyspnea, and bilateral lower extremity edema, especially on the right side that progressively worsened over 2 years. Initial diagnostic workup, including laboratory tests, color Doppler ultrasound, and echocardiography, revealed a large immobile mass attached to the RA wall. The patient underwent open‐heart surgery for mass debulking. The patient underwent chemotherapy. Unfortunately, during the final cycles of chemotherapy, she experienced worsening symptoms and progression of the mass, and she passed away after approximately 8 months due to cardiac arrhythmia. This case highlights the diagnostic and therapeutic challenges of PCL, particularly DLBCL. It underscores the importance of early recognition and multidisciplinary management in improving outcomes for this aggressive and rare malignancy.

**Summary**

- Primary cardiac lymphoma is one of the rarest primary malignancies of the heart.
- It accounts for < 0.5% of all primary cardiac tumors.
- Due to its rarity and nonspecific clinical manifestations, diagnosis is often challenging and frequently delayed until advanced stages of the disease.
- Therefore, increasing awareness of this condition is essential for timely identification and effective treatment.

## 1 Introduction

Primary cardiac lymphoma (PCL) is one of the rarest primary malignancies of the heart, exclusively involving the heart and its associated tissues without evidence of initial spread to other organs [1, 2]. This disease accounts for < 0.5% of all extra‐nodal lymphomas and < 2% of all primary cardiac tumors. Due to its rarity and nonspecific symptoms (such as dyspnea and chest pain), diagnosing PCL is challenging and often delayed until advanced stages of the disease. The clinical manifestations vary widely, ranging from heart failure to pericardial effusion and cardiac arrhythmias [3, 4]. Therefore, awareness of this disease is crucial for timely identification and effective treatment. PCLs are predominantly of the diffuse large B‐cell lymphoma (DLBCL) type, an aggressive form of non‐Hodgkin's lymphoma [5]. This disease is more commonly observed in immunocompromised patients, such as those with HIV or individuals undergoing organ transplantation, although cases have also been reported in immunocompetent patients [6]. The exact cause of PCL remains unknown, but it is likely associated with immune system dysfunction. Histologically, these tumors primarily develop in the myocardial and epicardial tissues and may also involve parts of the cardiac conduction system or heart valves. This involvement could explain symptoms such as atrioventricular (AV) block and atrial flutter [7]. The clinical manifestations of PCL vary depending on the location of cardiac involvement. In many cases, the disease primarily affects the right side of the heart, including the right atrium and right ventricle [8]. The most common symptoms include heart failure, pericardial effusion, and cardiac arrhythmias. Tumor masses can infiltrate the cardiac conduction system, leading to AV block, or other rhythm disturbances. Some patients may also present with systemic symptoms such as fever, night sweats, weight loss, and generalized weakness, consistent with systemic lymphomas' clinical features [9, 10]. The diagnosis of PCL is highly challenging due to its nonspecific symptoms and rarity. Various imaging modalities, including transesophageal echocardiography (TEE), computed tomography (CT), and magnetic resonance imaging (MRI), serve as key tools for detecting this condition [11]. Also, performing a biopsy at an early stage of the disease plays a crucial role in improving prognosis [12]. The prognosis of PCL is generally poor. The high mortality rate associated with PCL is not only due to its aggressive nature but also because of delayed diagnosis and treatment initiation [13]. The treatment of PCL typically begins with chemotherapy (includes the R‐CHOP chemotherapy regimen), as surgical intervention is rarely an option due to the complex location of tumor masses and the high risk of surgical procedures [14]. Since limited data are available on PCL and most clinical information is derived from case reports, further research is essential to gain a better understanding of its pathology, causes, and effective treatments. There is a pressing need to develop more efficient strategies for early diagnosis, appropriate treatment, and improved patient survival. Presenting a rare case of PCL can contribute to expanding current clinical knowledge and provide valuable insights for managing this uncommon disease. In this case report study, we examine a rare case of PCL in a 61‐year‐old female patient.

## 2 Case Presentation

**FIGURE 1:**
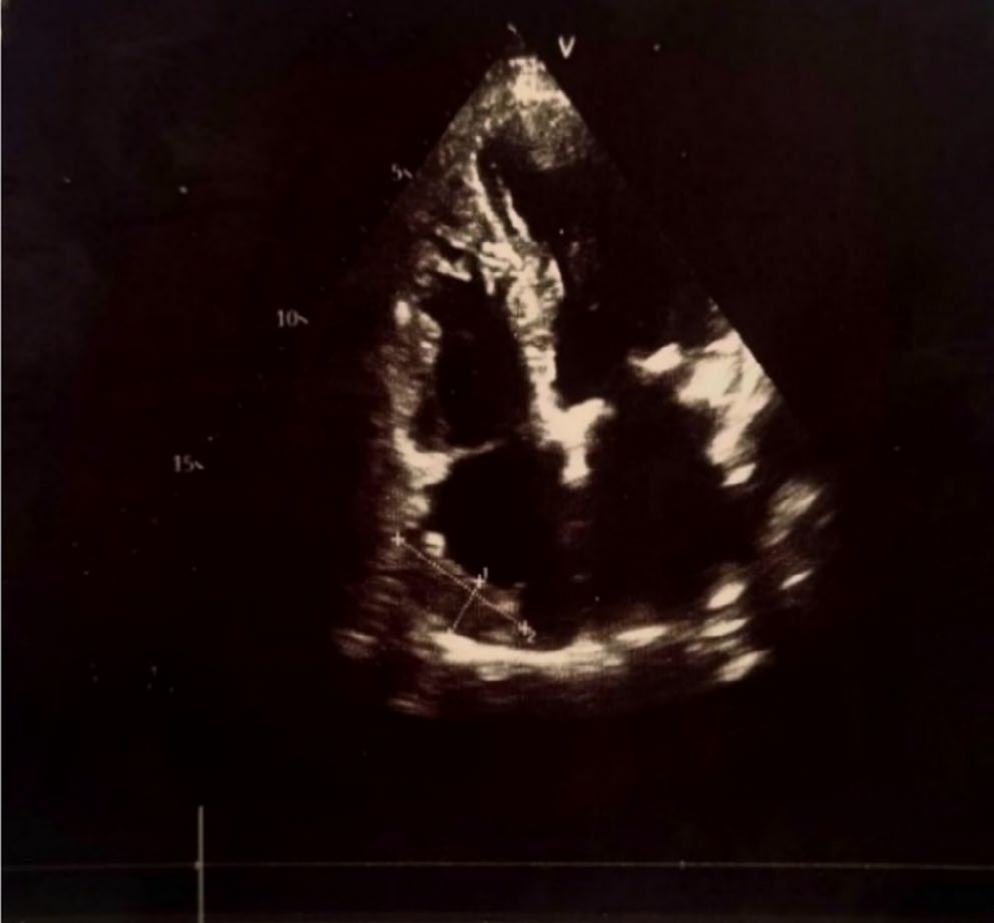
Transthoracic echocardiography showing a large intracardiac mass located in the right atrium.

A 61‐year‐old woman from Mazandaran, Iran, with a past medical history of hypertension, presented with weakness and bilateral lower extremity edema, especially on the right side. She had no symptoms of night sweats, fever, or weight loss. The patient's first symptoms began in June 2021 and persisted despite symptomatic management. Initial evaluations were standard, including vital signs, laboratory tests (CBC, RFT, LFT, TFT, electrolytes), lower limb color Doppler ultrasonography, ECG, and echocardiography (left ventricular ejection fraction [LVEF]: 55% with moderate left ventricular hypertrophy [LVH]). As symptoms persisted and worsened approximately over 2 years, the patient developed a shortage of breath (functional class II dyspnea), orthopnea, and increased lower limb edema. All of the examinations were repeated two times, and they were normal except for echocardiography, which reported that EF was 55% and a huge right atrial mass (87 × 53 mm) with right ventricular involvement (Figure 1). TEE confirmed a large immobile mass (87 × 53 mm) attached to the right atrial wall, extending to the orifice of the inferior vena cava (IVC), suggestive of myxoma. Left and right ventricular size and systolic function were normal, with a global EF of 55%, moderate LVH, mild to moderate tricuspid regurgitation (TR), moderate functional tricuspid stenosis (TS), and normal pulmonary artery systolic pressure (PAPs: 33 mmHg). Given these findings, the patient was referred for cardiac surgery. In May 2024, the patient underwent open‐heart surgery and a mass debulking procedure.

### 2.1 Diagnostic Workup

The last normal echocardiography was performed 34 months prior to symptom deterioration, and the initial abnormal transthoracic echocardiography (TTE) was performed in April 2024, which revealed a large right atrial mass with evidence of right ventricular invasion, raising significant concern for an intracardiac malignancy. A subsequent confirmatory TTE demonstrated the presence of an extensive intracardiac mass occupying the right atrium. Given the size and location of the lesion, the patient underwent open cardiac surgery with partial debulking of the mass.

**FIGURE 2:**
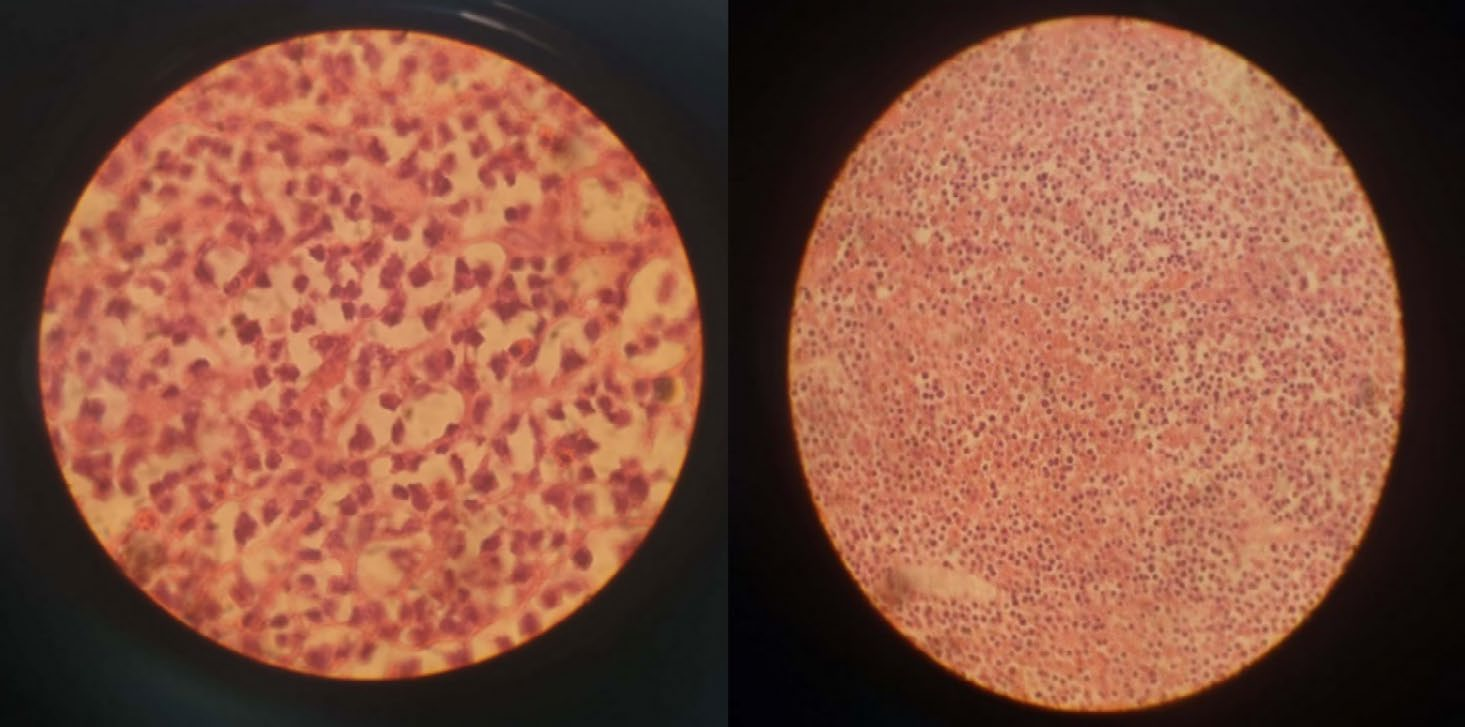
Cardiac mass shows diffuse proliferation of atypical lymphoid cells.

Histopathological examination of the excised tissue identified a high‐grade malignant neoplasm consistent with anaplastic large cell lymphoma (ALCL), a rare and aggressive subtype of non‐Hodgkin lymphoma (Figure 2). But immunohistochemical staining demonstrated diffuse positivity for CD20, CD19, PAX8, BCL2, and BCL6. Additional markers, including CD5, CD4, CD8, CD43, CD15, and MUM1, were also positive. Conversely, the tumor cells were negative for CD10, CD23, CD30, ALK, and CyclinD1. The Ki‐67 proliferation index was markedly elevated at 90%, supporting a highly proliferative and aggressive phenotype. Finally, IHC analysis confirmed the final diagnosis of DLBCL in the patient.

Postoperative echocardiography showed a mildly enlarged right atrium and moderate left atrial enlargement, with a persistent large mass (measuring 45 × 17 mm) attached to the right atrial free wall. The right ventricle was normal in size but showed mild systolic dysfunction. The LVEF was preserved at 55%. Additional findings included up to moderate TR, no TS, a pulmonary artery systolic pressure (PAPs) of 45 mmHg, and mild to moderate pulmonary hypertension. Moderate LVH was also noted (a comparative summary of preoperative and postoperative echocardiographic findings is presented in Table 1). Following surgical intervention, the patient's clinical condition showed partial improvement. Symptoms such as dyspnea and lower limb edema decreased, though they did not resolve entirely. Subsequently, the patient was referred to the oncology department at Shahid Rajaee Hospital in Babolsar for further management and continuation treatment.

**TABLE 1:** Comparative echocardiographic findings: preoperative versus postoperative and post‐chemotherapy (echocardiographic evaluations of the patients were independently performed by three different operators at the designated time intervals).

Parameter	Preoperative echocardiography	Postoperative/pre‐chemotherapy echocardiography	Post‐chemotherapy echocardiography
Left ventricle (LV)	Normal size and preserved function	Preserved systolic function, EF: 50%–55%	Preserved systolic function, EF: 50%–55%
Right ventricle (RV)	Normal size	Normal size, mild systolic dysfunction	Normal size and function
Left atrium (LA)	Mild bilateral enlargement	Moderate enlargement	Moderate enlargement
Right atrium (RA)	Large mass occupying the RA	Persistent mass (45 × 17 mm) attached to RA free wall; mild enlargement	Persistent mass (32 × 27 mm) attached to RA free wall; with compression effect to IVC, mild enlargement
Tricuspid valve	Moderate regurgitation (TR), No stenosis (Ts)	Up to moderate TR, no Ts	Moderate TR No Ts
Pulmonary artery pressure	PAP: 40 mmHg Mean PAP: 25 mmHg	PAPs: 45 mmHg, mild to moderate pulmonary hypertension	PAPs: 40 mmHg Mean PAP: 25 mmHg
LV hypertrophy	Mild LVH	Moderate LVH	Mild LVH
Other notable findings	Mass compressing IVC (3.2 × 2.7 cm)	Clinical improvement post‐surgery; symptoms partially resolved	—

### 2.2 Treatment and Outcome

By the time the diagnosis was confirmed, she had received eight cycles of chemotherapy with vincristine, doxorubicin, cyclophosphamide, rituximab, and prednisolone (R‐CHOP regimen) every 2 weeks. She completed her chemotherapy without any immediate adverse effects in December 2024. However, during the final stages of treatment, the patient experienced increasing dyspnea and recurrent bilateral lower limb edema. The patient was re‐evaluated with echocardiography, chest, and abdominopelvic CT scans, and the findings are outlined below.

**FIGURE 3:**
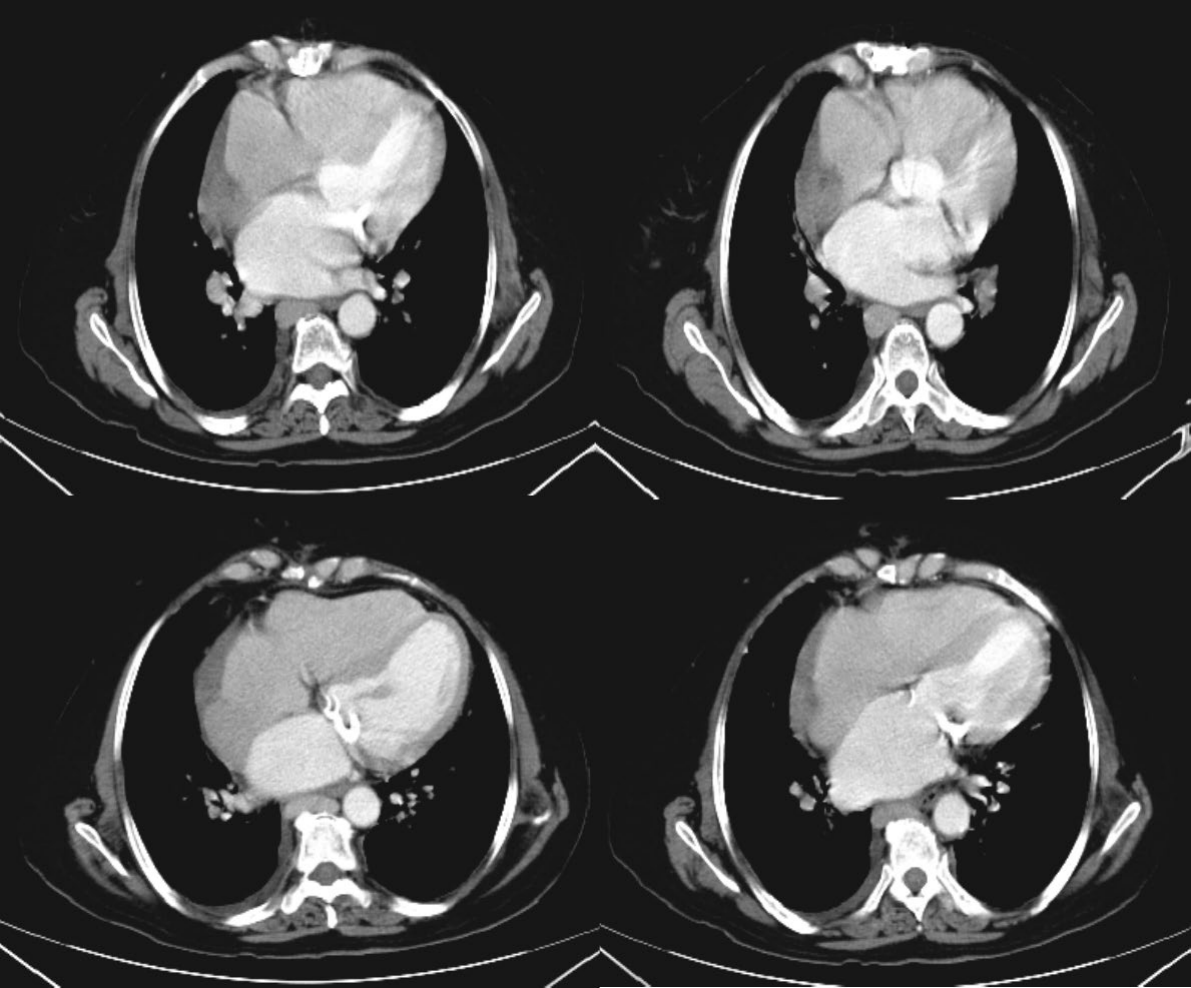
Spiral chest CT scan with contrast: images demonstrate an increase in the size of the mass in the right atrium.

Follow‐up TTE revealed a normal‐sized LV with preserved systolic function and an estimated EF of 50%–55%. Mild LVH was observed. The RV was normal in both size and function. The atria showed mild bilateral enlargement, with a left atrial volume index of 37 cc/m^2^ and a RA area of 19 cm^2^. A large, well‐defined mass measuring approximately 3.2 × 2.7 cm was noted attached to the RA free wall, exerting compressive effects on the IVC. Valvular assessment showed moderate TR without evidence of TS. The tricuspid regurgitant gradient was measured at 35 mmHg, and pulmonary artery pressure (PAP) was estimated at 40 mmHg. The pulmonary valve showed mild to moderate pulmonary insufficiency without pulmonary stenosis, and the mean pulmonary artery pressure was recorded at 25 mmHg (Table 1). CT scan of the chest revealed a hypo‐vascular soft tissue mass measuring 92 × 63 × 34 mm in the right pericardial region, suggestive of a progressive cardiac neoplasm (Figure 3). No additional abnormalities were noted on CT imaging.

Despite completion of chemotherapy, based on follow‐up CT scan demonstrated a slight increase in the size of the cardiac mass, indicating a poor therapeutic response to chemotherapy. Ten days following the final chemotherapy session, the patient presented to the emergency department with acute worsening of dyspnea, marking a significant clinical deterioration.

### 2.3 Emergency Department Presentation

The patient had cardiac arrhythmia, AF rhythm (heart rate: 95 bpm) (Figure 4), hypotension (blood pressure: 80/50 mmHg), and oxygen saturation was 80% in room air. Physical examination revealed distant and irregular heart sounds and bilateral crackles on lung sounds (Figure 5).

### 2.4 Diagnostic Workup in the Emergency Department

#### 2.4.1 Electrocardiogram (ECG)

**FIGURE 4:**
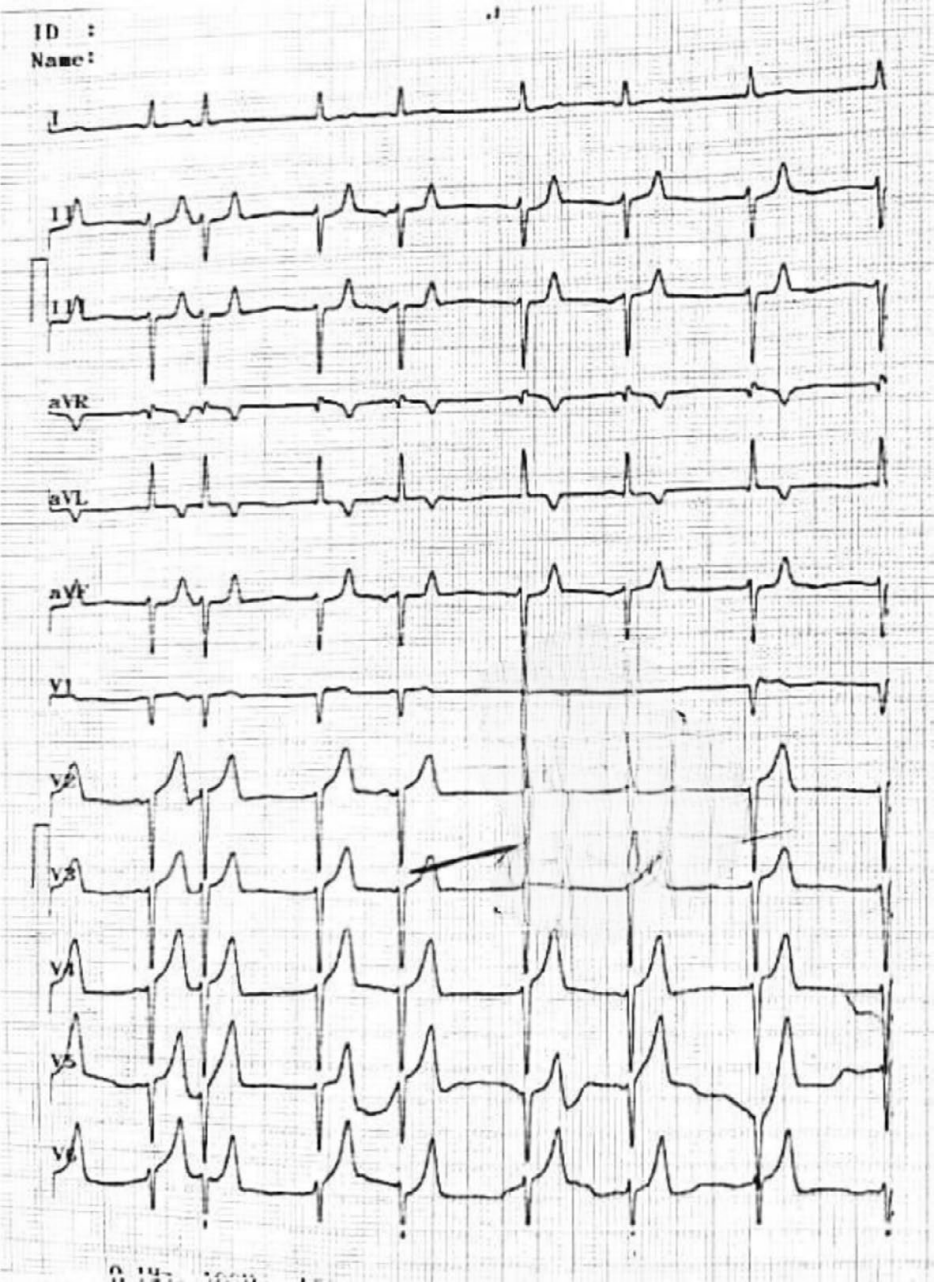
ECG (atrial fibrillation rhythm with approximately 95 bpm rate, poor R wave progression is noted in precordial leads).

#### 2.4.2 Chest X‐Ray

**FIGURE 5:**
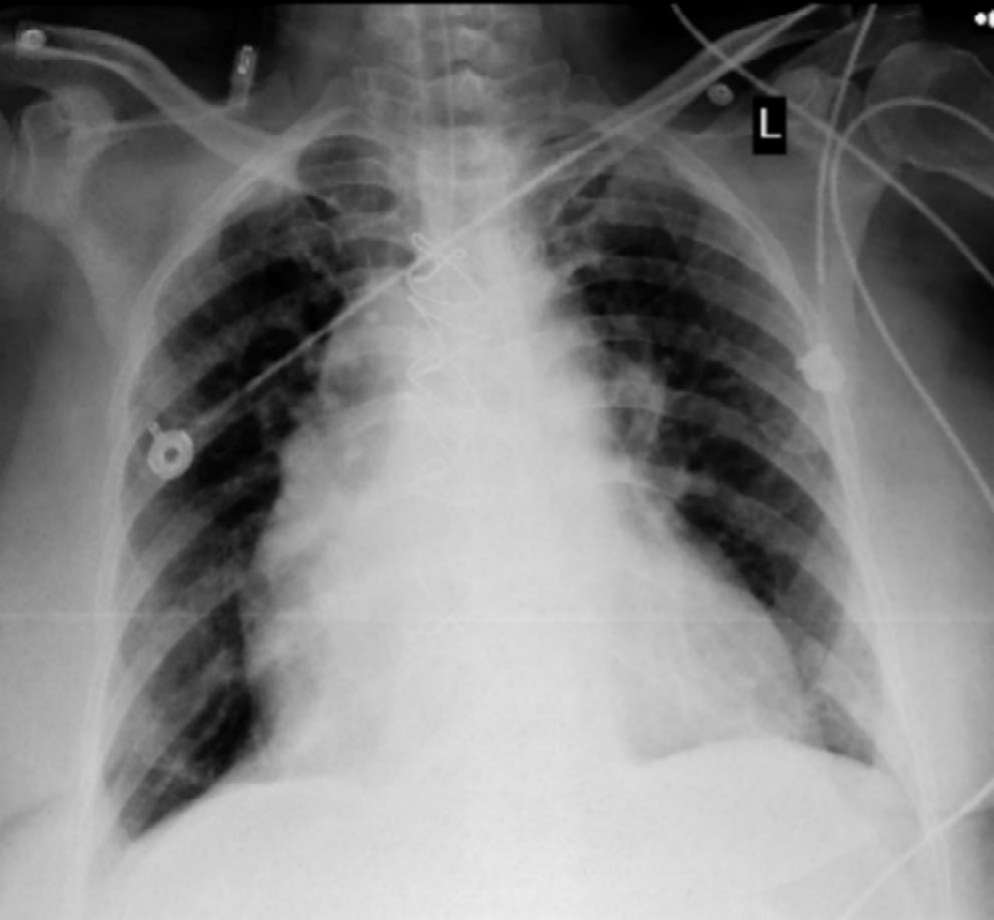
Chest X‐ray (portable chest): clear bilateral lung fields.

#### 2.4.3 Laboratory Test

She had positive troponin levels and hyperkalemia (*K* = 6). All other laboratory examinations were normal. Due to decreased oxygen saturation and reduced respiratory efforts, she was admitted to the intensive care unit and she was intubated. Despite initial stabilization, the patient's condition remained critical. Over the next few hours, she experienced cardiac arrest; resuscitation efforts were unsuccessful, and the patient expired approximately 8 months after initial diagnosis. Written informed consent for publication of this case report and accompanying images was obtained from the patient's legal representative.

## 3 Discussion

Primary cardiac lymphoma is an extremely rare malignancy. PCL presents with nonspecific and widespread symptoms, including cardiac symptoms such as heart failure, arrhythmias, pericardial effusion, or tamponade due to mass effect or infiltration, constitutional symptoms such as fever, night sweats, and weight loss (B symptoms), [7] and embolic phenomena such as systemic or pulmonary embolism due to tumor fragmentation [15]. This contributes to diagnostic challenges and a high mortality rate [15, 16, 17]. The aggressive clinical course of PCL parallels the catastrophic outcomes seen in acute aortic syndromes, such as ruptured dissecting aneurysms, where mortality approaches 94%–100% if untreated [18]. Both conditions exemplify the critical importance of early histopathologic diagnosis (e.g., via endomyocardial biopsy or aortic tissue analysis) and immediate intervention to avert fatal outcomes. Key diagnostic steps including:

1. Imaging: Echocardiography, CT, and MRI are essential for identifying cardiac masses. MRI, in particular, can help characterize the tumor's extent and involvement of surrounding structures [19].
2. Histopathology: Definitive diagnosis requires histopathological examination, often obtained via endomyocardial biopsy or pericardiocentesis. Anaplastic large cell lymphoma is characterized by large, pleomorphic cells expressing CD30 and often ALK (anaplastic lymphoma kinase) [20].
3. Immunohistochemistry: Staining for CD30, ALK, and other markers (e.g., EMA, T‐cell markers) is crucial for differentiating ALCL from other lymphomas or metastatic tumors [21].

In this case, the diagnosis was confirmed through an echocardiographic finding, then cardiac biopsy during debulking surgery, and immunohistochemistry findings. The more than 2‐year delay in diagnosis likely contributed significantly to the poor prognosis. Recent case reports—including a complex right atrial lymphoma—highlight the diagnostic value of minimally invasive transvenous endomyocardial biopsy (EMB) guided by intracardiac echocardiography (ICE) when atrial lesions are visualized on imaging. In these cases, ICE improves spatial localization of the bioptome, enhances sample accuracy, and reduces complication risks such as atrial perforation, tamponade, or arrhythmia. Transvenous approaches under TEE or ICE guidance have shown 100% diagnostic yield with relatively low morbidity compared to open surgical biopsies [22]. Nevertheless, when a cardiac mass is not amenable to transvenous sampling—due to size, location, or safety concerns—open cardiac biopsy during surgical debulking remains a definitive diagnostic strategy, as was performed in the present case. The aggressive clinical course of PCL parallels the catastrophic outcomes seen in acute aortic syndromes, such as ruptured dissecting aneurysms, where mortality approaches 94%–100% if untreated. Both conditions exemplify the critical importance of early histopathologic diagnosis (e.g., via endomyocardial biopsy or aortic tissue analysis) and immediate intervention to avert fatal outcomes [23]. There are multimodalities for the treatment of PCL, including chemotherapy, immunotherapy, radiotherapy, and surgery [24]. R‐CHOP chemotherapy (rituximab, cyclophosphamide, doxorubicin, vincristine, and prednisolone) is the predominant treatment option associated with improved survival [24, 25]. DA‐EPOCH‐R (dose‐adjusted etoposide, prednisolone, vincristine, cyclophosphamide, doxorubicin, and rituximab) is an alternative chemotherapy regimen in aggressive or refractory disease [26]. Due to tumor infiltration into the cardiac tissues and high surgical risks, surgical resection is not feasible [27]. In this case, the patient initially underwent surgery followed by eight courses of chemotherapy with rituximab, but unfortunately, she expired before radiation therapy. Overall survival following diagnosis was approximately 8 months. A recent systematic review study reported that the average survival among 188 cases was 2.2 years [25]. Generally, prognosis would depend on the extent of cardiac involvement, response to treatment, and presence of adverse prognostic factors. Also, this case highlights the necessity of multidisciplinary collaboration involving cardiology, oncology, cardiac surgery, and pathology to optimize diagnostic and therapeutic strategies in rare cardiac malignancies.

## 4 Conclusion

Primary cardiac lymphoma is a rare and aggressive malignancy that poses significant diagnostic and therapeutic challenges. A high index of suspicion, combined with advanced imaging and histopathological techniques, is essential for timely diagnosis. Multimodal treatment, including chemotherapy and targeted therapies, offers the best chance for disease control. However, the prognosis remains guarded, emphasizing the need for further research into novel therapeutic approaches and early detection strategies. This case underscores the importance of a multidisciplinary approach in managing rare cardiac malignancies.

## Author Contributions

**Danial Fazilat‐Panah:** conceptualization, funding acquisition, investigation, methodology, project administration, supervision. **Majid Nabipour:** data curation, investigation, methodology. **Seyed Reza Najafi:** data curation, investigation, writing – original draft, writing – review and editing. **Mina Heidarian:** data curation, investigation, writing – original draft, writing – review and editing.

## Conflicts of Interest

The authors declare no conflicts of interest.

## Data Availability

The data that support the findings of this study are available from the corresponding author upon reasonable request.
